# Diagnosis of Paediatric Extrapulmonary Tuberculosis by the MPT64 Antigen at a Tertiary Care Hospital

**DOI:** 10.7759/cureus.55688

**Published:** 2024-03-06

**Authors:** Ritu Kumari, Rakesh Kumar, Anita, Sweta Muni, Shailesh Kumar, Namrata Kumari

**Affiliations:** 1 Microbiology, Indira Gandhi Institute of Medical Sciences, Patna, IND

**Keywords:** extrapulmonary tuberculosis, mpt64 antigen, sensitivity, genexpert, paediatric

## Abstract

Background

Tuberculosis (TB) remains a global health concern, with India bearing a substantial burden. Paediatric TB, especially extrapulmonary TB (EPTB), presents unique diagnostic challenges due to its paucibacillary nature and the difficulty in obtaining suitable samples in children. Accurate and timely diagnosis is crucial to initiate appropriate treatment and mitigate disease spread. The MPT64 antigen test has shown promise in diagnosing TB, but its performance in paediatric EPTB remains underexplored. This study aimed to evaluate the diagnostic utility of the MPT64 antigen test in paediatric EPTB cases at a tertiary care hospital in India.

Methods

We conducted a prospective cross-sectional study at the Indira Gandhi Institute of Medical Sciences (IGIMS), a tertiary care hospital in India. A total of 250 paediatric participants, aged 0-18 years, with clinical suspicion of extrapulmonary tuberculosis (EPTB) were included. Diagnostic samples (e.g., tissue biopsies, pus, cerebrospinal fluid (CSF), and lymph node aspirates) were obtained, and tests including microscopy for acid-fast bacilli (AFB), mycobacterial cultures, GeneXpert MTB/RIF assay, and the TB Antigen MPT64 Rapid ICT Kit were performed. Sensitivity, specificity, positive predictive value (PPV), negative predictive value (NPV), and diagnostic accuracy of the MPT64 antigen test were calculated using culture and GeneXpert as reference standards.

Results

Among the 250 participants, 34 (13.6%) were confirmed to have EPTB. The MPT64 antigen test demonstrated a sensitivity of 70.6% and specificity of 92.1% in detecting EPTB cases. Mycobacterial cultures had the highest sensitivity (91.2%) and specificity (97.7%). GeneXpert showed a sensitivity of 70.6% and specificity of 93.9%. Overall diagnostic accuracy ranged from 88.7% for acid-fast bacteria (AFB) staining to 96.9% for mycobacterial cultures. The MPT64 antigen test had an area under the curve (AUC) of 0.814, indicating a good diagnostic accuracy.

Conclusion

The MPT64 antigen test demonstrates promising sensitivity and specificity for diagnosing paediatric EPTB, making it a valuable diagnostic tool, especially in resource-limited settings. However, mycobacterial cultures maintain the highest accuracy. Combining the MPT64 antigen test with other methods may enhance diagnostic capabilities.

## Introduction

Tuberculosis (TB) remains a significant global health concern, particularly affecting vulnerable populations, including children [1]. In the worldwide context of TB burden, India represents a significant share, contributing to approximately 26% of this global challenge. In India, the estimated incidence rate of TB stands at 188 cases per 100,000 population, and the mortality rate is notably high, reaching 37 deaths per 100,000 population, according to data from 2020. Alarmingly, children aged 0 to 14 years make up 11% of the total TB cases in the country [2].

Paediatric TB, often associated with extrapulmonary presentations, presents unique diagnostic challenges due to its diverse clinical manifestations and the paucibacillary nature of the disease. Extrapulmonary tuberculosis (EPTB) in children demands prompt and accurate diagnosis to ensure timely initiation of appropriate treatment and reduce morbidity and mortality [2]. In this context, the development and evaluation of diagnostic tools that enhance the detection of paediatric EPTB become pivotal [3].

Traditionally, the diagnosis of EPTB in children has heavily relied on conventional methods, such as acid-fast bacilli (AFB) staining, mycobacterial cultures, and clinical criteria. However, these approaches have limitations, including low sensitivity, delayed results, and the need for invasive procedures, especially when obtaining diagnostic samples from deep-seated sites [4,5]. Consequently, novel diagnostic strategies are urgently needed to improve the accuracy and efficiency of paediatric EPTB diagnosis [6,7,8,9].

One promising diagnostic approach is the use of antigen detection tests, which target specific *Mycobacterium tuberculosis* (MTB) antigens in patient samples [10]. Among these, the MPT64 antigen has shown potential as a valuable diagnostic marker for TB. The MPT64 antigen is a highly immunogenic protein secreted by MTB and has been reported to be present in a wide range of clinical specimens [11]. Its detection, particularly in clinical samples, can enhance diagnostic sensitivity and expedite the confirmation of paediatric EPTB cases [12,13].

In light of these challenges, the present study aimed to assess the sensitivity, specificity, positive predictive value (PPV), negative predictive value (NPV), and overall diagnostic accuracy of the MPT64 antigen-based kit in comparison to conventional diagnostic methods, including AFB staining, mycobacterial cultures, and GeneXpert.

## Materials and methods

Study design and area

This retrospective cross-sectional study was conducted at the Indira Gandhi Institute of Medical Sciences (IGIMS) located in Patna, Bihar, India. The study duration spanned one year and six months, commencing in January 2022 and concluding in June 2023.

Study participants and sample size

Participants eligible for inclusion were children between the ages of 0 to 18 years who were attending IGIMS, Patna, and presented with clinical suspicion of EPTB. All the cases of pulmonary TB and the patients who did not follow up after the initial evaluation were excluded from the study. With a confidence level of 95% (α = 0.05), a power of 80% (β = 0.20), and an estimated EPTB prevalence of 10% in children, a margin of error of 5% (0.05) was chosen and a minimum sample size of 223 was calculated. Taking a dropout rate of 10% into consideration, a sample size of 250 was estimated. The study included a total of 250 participants who met the specified criteria.

Diagnostic samples and laboratory tests

For patients presenting with superficial, palpable masses, enlarged lymph nodes, and pus-filled abscesses or lesions, the diagnostic material was collected using a 22-gauge needle attached to a syringe. During the collection procedure, minimal negative pressure was applied to minimize tissue damage and ensure sample quality. In cases where EPTB was suspected to affect the central nervous system, cerebrospinal fluid (CSF) samples were obtained for diagnostic purposes. CSF samples were collected through standard lumbar puncture procedures, ensuring aseptic techniques to prevent contamination. Tissue biopsy samples were collected from patients who presented with clinical signs and symptoms suggestive of EPTB and required a more invasive diagnostic approach. The biopsy procedure involved the surgical removal of a small tissue sample from the suspected site of infection, such as lymph nodes, organ lesions, or other relevant anatomical locations. These tissue biopsy samples were handled carefully to preserve their integrity and ensure minimal damage during the collection process.

The collected diagnostic material was subsequently smeared onto glass slides for further examination. These smears were used for cytological examination and Ziehl-Neelsen (ZN) staining. Cytological examination involved the microscopic analysis of cellular components within the smears. This examination helped assess the cellular characteristics of the collected material and identify any abnormal or pathological changes. Staining is a well-established laboratory technique used for the detection of AFB. In this process, the smears were stained using the ZN staining method, which stains AFB (including *MTB*) red against a blue background. This staining method is pivotal for identifying AFB in clinical specimens, aiding in the diagnosis of TB.

Mycobacterial cultures were performed on a Lowenstein-Jensen medium, following the standard protocols established by the Central Tuberculosis Reference Laboratory. These cultures aimed to provide a favorable environment for the growth of *MTB*. Positive cultures confirmed the presence of viable MTB bacilli in the diagnostic material. To obtain specimens for mycobacterial culture and GeneXpert MTB/RIF assay, a sterile saline solution was employed to rinse the needle and syringe used for sample collection. This ensured that the collected diagnostic material was appropriately prepared for further laboratory testing. The TB Antigen MPT64 Rapid ICT Kit was utilized in accordance with the manufacturer's instructions. This diagnostic kit employs monoclonal mouse antibodies against the MPT64 antigen, which is specific to *MTB*. The kit is based on an immunochromatographic test (ICT) principle and is designed to confirm the presence of MTB isolates in the collected diagnostic samples. It offers a rapid and specific diagnostic tool for TB.

All laboratory procedures were conducted within a biosafety class II cabinet, adhering to level two biosafety procedures to ensure the safety of laboratory personnel and prevent contamination.

Data collection and analysis

A preformed proforma was used to capture the demographic details and clinical history, and physical examination findings were recorded for each enrolled patient at the time of their initial presentation to IGIMS. Statistical analysis of the diagnostic test results was performed using IBM SPSS Statistics for Windows, version 20 (released 2011; IBM Corp., Armonk, New York, United States). The demographic and clinical characteristics of the study participants were summarized using descriptive statistics. This included calculating means, standard deviations, frequencies, and percentages where appropriate to describe the patient population. The diagnostic performance of the TB Antigen MPT64 Rapid ICT Kit was evaluated in terms of sensitivity, specificity, PPV, NPV, and diagnostic accuracy. These parameters were calculated using mycobacterial culture and GeneXpert results as reference standards.

Sensitivity represented the proportion of true-positive results among confirmed EPTB cases, while specificity indicated the proportion of true-negative results among non-EPTB cases. PPV was the probability that a positive test result corresponded to EPTB, and NPV was the probability that a negative test result ruled out EPTB. Diagnostic accuracy represented the overall correctness of the TB Antigen MPT64 Rapid ICT Kit in diagnosing EPTB. Subgroup analysis was conducted to investigate the influence of factors, such as age, gender, and sample type, on the diagnostic performance of the TB Antigen MPT64 Rapid ICT Kit. Sensitivity, specificity, PPV, NPV, and diagnostic accuracy were calculated within each subgroup to assess potential variations in test performance based on these factors.

Receiver operating characteristic (ROC) analysis and the calculation of the area under the curve (AUC) were performed to further assess the diagnostic accuracy of the TB Antigen MPT64 Rapid ICT Kit in paediatric patients suspected of having EPTB. The ROC curve was generated by plotting the true positive rate (sensitivity) against the false positive rate (1-specificity) at various threshold values. This curve illustrates the trade-off between sensitivity and specificity and helps identify the optimal cutoff point that maximizes diagnostic accuracy. The AUC was calculated to quantify the overall diagnostic accuracy of the TB Antigen MPT64 Rapid ICT Kit. The AUC value provides a single measure of the test's ability to discriminate between EPTB and non-EPTB cases. An AUC value of 1.0 indicates perfect discrimination, while 0.5 suggests no discriminatory power (random chance). A p-value of less than 0.05 was considered statistically significant.

Ethical considerations

Ethical approval for this study was obtained from the Institutional Review Committee of IGIMS (approval number: 1115/IEC/IGIMS/2022, dated: 22/07/2022). Patient confidentiality was rigorously upheld throughout the study, and informed consent was secured from the legal guardians of all participating children.

## Results

In our study, we analyzed the characteristics of paediatric patients with suspected EPTB who were attending the IGMIS in Patna, Bihar. The mean age of the participants was 11.16 years, with a minimum age of three years and maximum age of 17 years, reflecting the relatively young age of the study population. Regarding gender distribution, the study included 146 male participants (58.4%) and 104 female participants (41.6%). This distribution provides a representative sample of both genders in the study.

To obtain diagnostic samples, various sources were utilized. Tissue biopsy samples were taken from 84 participants (33.6%), while pus samples were collected from 83 participants (33.2%). CSF samples were obtained from 42 participants (16.8%), and lymph node aspirates were taken from 41 participants (16.4%). These diverse sample sources reflect the complexity and variability in diagnosing paediatric EPTB. In terms of the diagnosis of EPTB, our findings indicated that 34 participants (13.6%) were confirmed to have EPTB, while 216 participants (86.4%) did not have EPTB. These results highlight the importance of accurate diagnostic methods to confirm EPTB cases in children, given the potential challenges associated with diagnosis in this patient population (Table 1).

**Table 1:** Baseline characteristics of the study participants (n = 250). EPTB: extrapulmonary tuberculosis, CSF: cerebrospinal fluid

Characteristic	Frequency	%
Mean Age (years)	11.16 ± 4.03	
Gender		
Male	146	58.4
Female	104	41.6
Sample		
Tissue biopsy	84	33.6
Pus	83	33.2
CSF	42	16.8
Lymph node	41	16.4
EPTB		
Yes	34	13.6
No	216	86.4

We assessed diagnostic methods for paediatric patients with suspected EPTB, distinguishing those with confirmed EPTB (n = 34) from those without EPTB (n = 216). AFB staining showed 61.7% (n = 21) sensitivity in confirmed EPTB cases and 7.4% in non-EPTB cases (n = 16), indicating higher sensitivity in confirmed EPTB cases. Mycobacterial cultures yielded 91.2% positivity in EPTB-positive cases (n = 31) and 2.3% in EPTB-negative cases (n = 5), highlighting their diagnostic value. The GeneXpert MTB/RIF assay displayed 70.6% sensitivity in EPTB-positive cases (n = 24) but had a 6.5% positivity rate in non-EPTB cases (n = 14). The MPT64 antigen test had a sensitivity of 70.6% in EPTB-positive cases (n = 24) and 7.9% in non-EPTB cases (n = 17), indicating good sensitivity but slightly higher positivity in non-EPTB cases compared to the GeneXpert assay (Table 2).

**Table 2:** Comparison of diagnostic methods for paediatric EPTB. EPTB: extrapulmonary tuberculosis, AFB: acid-fast bacilli, MTB/RIF: Mycobacterium tuberculosis/rifampicin

Diagnostic methods	EPTB	
	Yes (n = 34) Frequency (%)	No (n = 216) Frequency (%)
AFB staining		
Positive	21 (61.7)	16 (7.4)
Negative	13 (38.2)	200 (92.6)
Mycobacterial cultures		
Positive	31 (91.2)	5 (2.3)
Negative	3 (8.8)	211 (97.7)
GeneXpert MTB/RIF assay		
Positive	24 (70.6)	14 (6.5)
Negative	10 (29.4)	202 (93.5)
MPT64 antigen test		
Positive	24 (70.6)	17 (7.9)
Negative	10 (29.4)	199 (92.1)

In comparing the diagnostic performance of the MPT64 antigen test with other tests for paediatric patients with suspected EPTB, 'confirmed EPTB' was defined as any positive result in culture or GeneXpert MTB/RIF assay, while 'non-EPTB' was defined as negative results for these tests. This comprehensive definition ensures that cases classified as 'confirmed EPTB' exhibit evidence of *MTB* through multiple diagnostic criteria, contributing to a more robust evaluation of the MPT64 antigen test in comparison to other diagnostic methods.

In our study, several noteworthy findings emerged. First, when considering sensitivity, both the MPT64 antigen test and the GeneXpert MTB/RIF assay displayed similar levels, at 70.6% and 70.6%, respectively, while mycobacterial cultures exhibited the highest sensitivity at 91.2%. AFB staining lagged behind with a sensitivity of 61.7%. In terms of specificity, mycobacterial cultures led the way with an impressive 97.7%, closely followed by AFB staining at 92.6%, the MPT64 antigen test at 92.1%, and the GeneXpert MTB/RIF assay at 93.9%. For PPV, mycobacterial cultures had the highest at 84.9%, with the MPT64 antigen test and GeneXpert MTB/RIF assay having PPVs of 56.2% and 62.4%, respectively, and AFB staining at 54.4%. Regarding NPV, mycobacterial cultures excelled with 98.7%, followed closely by AFB staining at 94.4%, the MPT64 antigen test at 95.6%, and the GeneXpert MTB/RIF assay at 95.7%. Overall diagnostic accuracy revealed that mycobacterial cultures achieved the highest accuracy at 96.9%, followed by the GeneXpert MTB/RIF assay at 91%, the MPT64 antigen test at 89.4%, and AFB staining at 88.7% (Table 3).

**Table 3:** Diagnostic test performance of diagnostic methods for paediatric EPTB. a: Under the nonparametric assumption, b: Null hypothesis: true area = 0.5 EPTB: extrapulmonary tuberculosis, CI: confidence interval, PPV: positive predictive value, NPV: negative predictive value, AFB: acid-fast bacilli, MTB/RIF: Mycobacterium tuberculosis/rifampicin

Diagnostic tests	Sensitivity % (95% CI)	Specificity % (95% CI)	PPV % (95% CI)	NPV % (95% CI)	Diagnostic accuracy % (95% CI)
AFB staining	61.7 (43.6 to 77.8)	92.6 (88.3 to 95.7)	54.4 (41.0 to 67.2)	94.4 (91.7 to 96.3)	88.7 (84.2 to 92.4)
Mycobacterial cultures	91.2 (76.3 to 98.1)	97.7 (94.7 to 99.2)	84.9 (70.2 to 93.1)	98.7 (96.3 to 99.6)	96.9 (93.9 to 98.7)
GeneXpert MTB/RIF assay	70.6 (52.5 to 84.9)	93.9 (90.0 to 96.6)	62.4 (48.8 to 74.2)	95.7 (93.0 to 97.4)	91.0 (86.9 to 94.2)
MPT64 antigen test	70.6 (52.5 to 84.9)	92.1 (87.7 to 95.4)	56.2 (43.6 to 68.0)	95.6 (92.9 to 97.4)	89.4 (85.0 to 93.0)

**Figure 1:**
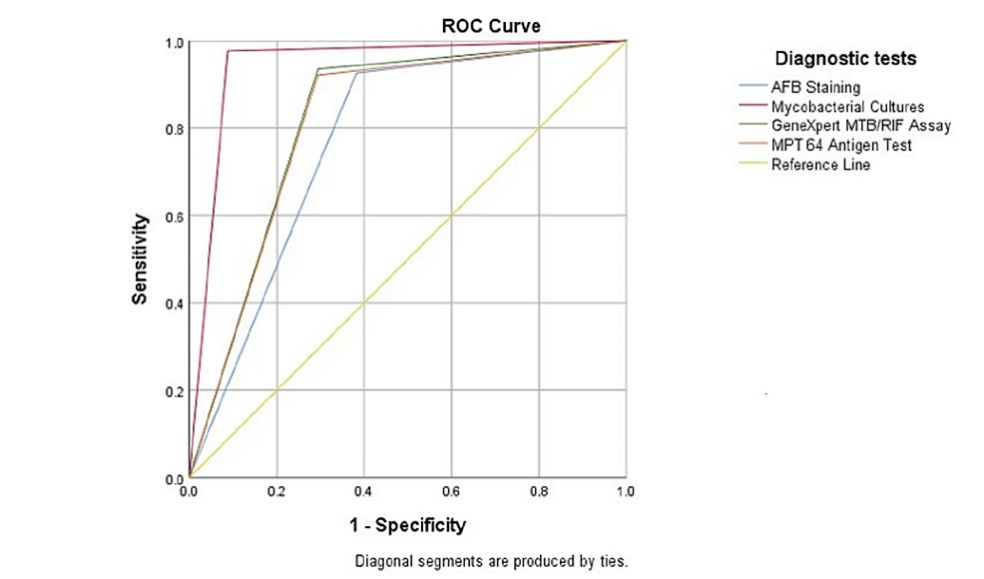
Area under the curve (AUC) for the diagnostic accuracy of various diagnostic tests.

The diagnostic accuracy of various tests for paediatric patients suspected of having EPTB was assessed, and the results showed significant differences among the methods (p < 0.001). Mycobacterial cultures exhibited the highest AUC at 0.944, followed closely by the MPT64 antigen test and the GeneXpert MTB/RIF assay, with AUC values of 0.814 and 0.821, respectively. AFB staining had the lowest AUC at 0.772. These findings indicate that mycobacterial cultures had the most robust diagnostic accuracy, followed closely by the MPT64 antigen test and the GeneXpert MTB/RIF assay, while AFB staining showed comparatively lower diagnostic accuracy in the context of paediatric EPTB diagnosis (Table 4 and Figure 1).

**Table 4:** Area under the curve (AUC) for the diagnostic accuracy of various diagnostic tests. p value was considered significant at p value<0.05. AFB: acid-fast bacilli, MTB/RIF: Mycobacterium tuberculosis/rifampicin

Diagnostic tests	Area	Std. error^a^	P value^b^	95% confidence interval	
				Lower limit	Upper limit
AFB staining	0.772	0.052	0.000	0.670	0.874
Mycobacterial cultures	0.944	0.029	0.000	0.888	1.000
GeneXpert MTB/RIF assay	0.821	0.048	0.000	0.727	0.914
MPT64 antigen test	0.814	0.048	0.000	0.720	0.907

The main findings of our study indicate that the MPT64 antigen test showed variable sensitivity and specificity when stratified by age, gender, and sample type. In the age category, the test demonstrated 33.3% sensitivity in the zero to five years' group, 70.0% in the six to 10 years' group, and 66.7% in the 11-18 years' group. In terms of gender, the test exhibited 76.2% sensitivity in males and 61.5% in females. Regarding sample types, the test had 90.9% sensitivity for tissue biopsy, 70.0% for pus, 42.9% for CSF, and 66.7% for lymph node samples. Specificity remained relatively consistent across these categories. The PPV ranged from 34.9% to 76.9%, while the NPV ranged from 90.8% to 98.6%. Overall diagnostic accuracy ranged from 82.9% to 92.9% across the different subgroups. These findings emphasize the importance of considering patient demographics and sample type when interpreting MPT64 antigen test results for the diagnosis of paediatric EPTB (Table 5).

**Table 5:** Subgroup analysis by age, gender, and sample for the MPT64 diagnostic test. CSF: cerebrospinal fluid

MPT64 antigen test	Sensitivity % (95% CI)	Specificity % (95% CI)	PPV % (95% CI)	NPV % (95% CI)	Diagnostic accuracy % (95% CI)
Age					
0-5 years	33.3 (0.8 to 90.6)	94.4 (72.7 to 99.9)	46.2 (6.7 to 91.2)	90.8 (81.6 to 95.7)	86.8 (65.0 to 97.4)
6-10 years	70.0 (34.8 to 93.3)	90.4 (81.2 to 96.1)	51.1 (31.6 to 70.2)	95.5 (89.1 to 98.2)	87.9 (78.9 to 94.0)
11-18 years	66.7 (44.7 to 84.4)	92.8 (86.8 to 96.7)	57.0 (39.9 to 72.5)	95.1 (91.7 to 97.2)	89.5 (83.5 to 94.0)
Gender					
Male	76.2 (52.8 to 91.8)	91.2 (84.8 to 95.5)	55.3 (40.1 to 69.5)	96.4 (92.6 to 98.3)	89.3 (83.2 to 93.8)
Female	61.5 (31.6 to 86.1)	93.4 (86.2 to 97.5)	57.1 (35.5 to 76.4)	94.4 (89.5 to 97.1)	89.4 (81.9 to 94.6)
Sample					
Tissue biopsy	90.9 (58.7 to 99.8)	93.2 (84.7 to 97.7)	65.5 (44.4 to 81.9)	98.6 (91.7 to 99.8)	92.9 (85.1 to 97.3)
Pus	70.0 (34.8 to 93.3)	90.4 (81.2 to 96.1)	51.1 (31.6 to 70.2)	95.5 (89.1 to 98.2)	87.9 (78.9 to 94.0)
CSF	42.9 (9.9 to 81.6)	88.6 (73.3 to 96.8)	34.9 (13.2 to 65.3)	91.6 (85.0 to 95.4)	82.9 (68.1 to 92.7)
Lymph node	66.7 (22.3 to 95.7)	97.1 (85.1 to 99.9)	76.9 (30.8 to 96.2)	95.3 (86.8 to 98.5)	93.3 (81.0 to 98.7)

## Discussion

The findings of our study provide valuable insights into the diagnostic landscape of paediatric EPTB and offer significant implications for clinical practice. Paediatric EPTB is a challenging clinical entity due to its diverse presentations, non-specific symptoms, and difficulties in obtaining diagnostic samples. In this discussion, we will delve into the key findings of our study and their implications, address the strengths and limitations of the research, and suggest future directions in paediatric EPTB diagnosis.

Age and gender disparities

Our study revealed intriguing age-related disparities in the performance of the diagnostic methods, particularly the MPT64 antigen test. The test exhibited lower sensitivity in the zero to five years' age group (33.3%) compared to the six to 10 years' (70.0%) and 11-18 years' (66.7%) groups. This variation might be attributed to differences in immune response, disease manifestation, or sample characteristics among these age groups. Clinicians should be aware of these age-related nuances when interpreting MPT64 antigen test results. Furthermore, we observed differences in sensitivity between male (76.2%) and female (61.5%) participants, which might be influenced by hormonal or immunological factors. These gender-related variations warrant further investigation to elucidate their underlying mechanisms.

Sample source impact

The type of diagnostic sample significantly influenced the diagnostic performance of the MPT64 antigen test. The test demonstrated excellent sensitivity (90.9%) in tissue biopsy samples, making it a robust choice for cases where this sample type is accessible. However, its sensitivity dropped notably in pus (70.0%), CSF (42.9%), and lymph node aspirates (66.7%). These findings underscore the need for tailored diagnostic strategies based on the sample source. In addition, the lower sensitivity in CSF samples underscores the challenges in diagnosing paediatric tuberculous meningitis, a severe form of EPTB. In a study by Purohit et al., fine-needle aspiration cytology (FNAC) has demonstrated a lack of specificity in different contexts, where conditions, such as sarcoidosis, non-tuberculous mycobacteria, and various granulomatous diseases, may imitate TB [14]. Sypabekova et al. showed that for sputum samples, the specificity and sensitivity of MPT64 were 100% and 76%, respectively, and for serum samples, they were 100% and 88%, respectively [15]. The study in Jorstad et al. found a sensitivity of the MPT64 test of 100% for paediatric TB adenitis [16]. In a study by Kohli et al., it has been demonstrated that GeneXpert exhibits a similar, albeit slightly reduced, level of sensitivity when compared to *MTB* culture in the context of different EPTB samples [17].

Comparative diagnostic performance

Comparing the diagnostic methods, mycobacterial cultures emerged as the gold standard, exhibiting the highest sensitivity (91.2%) and specificity (97.7%). However, the prolonged turnaround time for culture results limits its immediate clinical utility. The GeneXpert MTB/RIF assay and the MPT64 antigen test demonstrated similar sensitivities (70.6%) and specificity (92.1% and 93.9%, respectively). The advantage of the GeneXpert MTB/RIF assay lies in its rapid results, which can expedite treatment initiation, especially in resource-limited settings. The MPT64 antigen test, despite its slightly lower specificity, offers a quicker alternative when culture facilities are unavailable. Therefore, its performance characteristics and rapidity make it a valuable tool for initial screening, albeit with some caution regarding specificity. Qin et al. revealed a significant difference (P < 0.01) in detecting MPT64 protein levels in the culture filtrates between group A (non-TB mycobacterium) and group B (*MTB*) and demonstrated a high sensitivity of 86.3% and specificity of 88.5% [18]. Purohit et al. have also highlighted the effectiveness of the MPT64 test for diagnosing paediatric EPTB [19]. The sensitivity of MPT64 varied between 67% and 100% in previous studies by Baba et al., Tadele et al., and Purohit et al. [14,20,21].

AUC analysis

The AUC analysis reinforced the diagnostic accuracy hierarchy established in our study. Mycobacterial cultures had the highest AUC (0.944), closely followed by the MPT64 antigen test (0.814) and the GeneXpert MTB/RIF assay (0.821), while AFB staining had the lowest AUC (0.772). This statistical approach confirmed the diagnostic superiority of mycobacterial cultures and the promising performance of the MPT64 antigen test and the GeneXpert MTB/RIF assay. The studies by Mustafa et al. and Hunter et al. have shown the enhanced effectiveness of MTP64 in comparison to *MTB* culture and GeneXpert can be ascribed to the fact that EPTB often presents with a low bacillary load, and it underscores the possible involvement of antigen accumulation as a crucial element in the pathogenesis of EPTB [22,23].

Clinical implications

Our findings have direct clinical implications for the diagnosis of paediatric EPTB. Clinicians must be aware of the age-related differences in test performance and the impact of the sample source. While mycobacterial cultures remain the gold standard, they may not always be feasible due to the lengthy turnaround time. The GeneXpert MTB/RIF assay can serve as a rapid and reliable alternative, especially for critically ill patients. The MPT64 antigen test, despite its slightly lower specificity, can be a valuable initial screening tool, expediting diagnosis and treatment initiation, particularly in settings with limited laboratory resources. Currently available nucleic acid amplification test (NAAT)-based tests offer rapid results and the added benefit of universal drug testing. However, drawbacks, such as their high cost and the need for a continuous power source for maintenance, contribute to the challenges, particularly in resource-constrained settings [24]. In addition to its diagnostic performance, the MPT64 antigen test presents a promising solution to the challenges associated with traditional diagnostic methods. With its rapid turnaround time, cost-effectiveness, and user-friendly application, the MPT64 antigen test addresses critical issues in resource-limited settings, offering a valuable tool for the timely and accurate diagnosis of paediatric EPTB.

Limitations

A notable strength of our study is its focus on paediatric EPTB, an area with limited research. We included a diverse patient population and evaluated multiple diagnostic methods. However, the study is not without limitations. It is a single-center study, which may limit the generalizability of the findings. The sample size, while sufficient for our analysis, may not capture rare manifestations of EPTB. In addition, variations in sample collection and processing techniques may introduce bias.

## Conclusions

Our study underscores the importance of accurate diagnostic methods for paediatric EPTB, considering the challenges of diagnosing this condition in children. Mycobacterial cultures demonstrated the highest diagnostic accuracy, followed by the GeneXpert MTB/RIF assay and the MPT64 antigen test, while AFB staining lagged behind. The MPT64 antigen test displayed variable sensitivity and specificity, with performance varying by age, gender, and sample type. These findings emphasize the need for tailored diagnostic approaches in paediatric EPTB cases and suggest that the MPT64 antigen test can be a valuable tool in the diagnostic arsenal, particularly when interpreted in context.
